# Grieving a disrupted biography: an interpretative phenomenological analysis exploring barriers to the use of mindfulness after neurological injury or impairment

**DOI:** 10.1186/s40359-021-00628-0

**Published:** 2021-08-24

**Authors:** K. A. Finlay, J. H. Hearn, A. Chater

**Affiliations:** 1 School of Psychology and Clinical Language Sciences, University of Reading, Reading, Berks, RG6 7BE UK; 2 Department of Psychology, Manchester Metropolitan University, Brooks Building, 53 Bonsall Street, Manchester, M15 6GX UK; 3 Institute for Sport and Physical Activity Research (ISPAR), Centre for Health, Wellbeing and Behaviour Change, University of Bedfordshire, Polhill Avenue , Bedford, MK41 9EA UK

**Keywords:** Interview, Qualitative, Mental processes, Meditation, Grief, Neurological rehabilitation

## Abstract

**Background:**

Mindfulness has demonstrated strong utility for enhancing self-management and health outcomes in chronic illness. However, sensation-focused mindfulness techniques may not be appropriate for clinical populations with neurological injury. This study aimed to identify how expert mindfulness teachers with sensory loss/impairment naturalistically adapt and experience mindfulness. We aimed to highlight the rationale for and barriers to mindfulness practice when living with sensory loss.

**Methods:**

A qualitative, semi-structured interview design was used, analysed via Interpretative Phenomenological Analysis (IPA). Eight (5 females, 3 males) mindfulness teachers with neurological injury were recruited via a national registry of Mindfulness for Health teachers. Interviews (range: 50–93 min) were completed, transcribed verbatim and analysed idiographically for descriptive, linguistic and conceptual themes, before a cross-case analysis was completed.

**Results:**

Two superordinate themes were identified: (1) Overcoming a disrupted biography; and (2) Proactive self-management. These themes considered the challenge of reconciling, through grief, a past health status with the present reality of living with sensory loss due to Spinal Cord Injury, Multiple Sclerosis or Functional Neurological Disorder. Mindfulness was experienced as a method by which proactive choices could be made to maintain control and autonomy in health, reducing perceptions of suffering, psychological distress, cognitive reactivity and rumination.

**Conclusions:**

Mindfulness was found to support the self-management of health after neurological injury/impairment. Mindfulness meditation presented an initial challenge as trauma and grief processes were (re-)activated during mindfulness sessions. However, mindfulness was found to support the resolution of these grief processes and encourage adaptive approach-based coping and acceptance of health and neurological impairment/injury.

## Background

Mindfulness is widely employed as an adjunctive strategy for health self-management in people with chronic health conditions [1]. Core mindfulness techniques include increased breath awareness and focusing on the present moment by paying attention to physical sensations, therefore minimising maladaptive cognitive reactivity [2]. Mindfulness has been implicated as a stress-buffering strategy which can reduce the psychological distress, depression, pain and worry associated with living with chronic illness [3–5]. Mindfulness-based practices employ strategies that promote acceptance of (ill-)health through non-judgemental observation of thoughts and feelings [1]. This can interrupt cycles of rumination and catastrophizing linked with poor quality of life and reduced psychological wellbeing in people with chronic health conditions [6, 7]. The utility of mindfulness for chronic illness management is wide-ranging: mindfulness has been shown to have positive psychological and physical health benefits for a significant number of chronic health conditions, including Spinal Cord Injury (SCI) [8], Multiple Sclerosis (MS) [9], Chronic Obstructive Pulmonary Disease [10], Parkinson’s Disease [11], stroke and traumatic brain injury [12]. As a consequence, mindfulness has been increasingly recommended for consideration in the rehabilitation and management of chronic illness [13].

Though evidence for mindfulness as supportive for self-management of chronic conditions is strong, previous research has suggested that specific barriers to mindfulness may exist if mindfulness is employed after traumatic injury or in conditions with sensory loss, such as Spinal Cord Injury [8] and Multiple Sclerosis [14]. The barriers may reflect the focus of core mindfulness techniques (such as body scanning and mindful walking) on sensory awareness [2, 15]. Certainly, barriers are both physical and psychological: physically, they include difficulties managing disability-related factors during meditation, such as the expectation of supine meditation and inclusion of the term ‘mindful walking’ in standardised mindfulness interventions, causing perceptions of inaccessibility or physical risk [14]. In association, the sensation-focused language used in Mindfulness-based Stress Reduction (MBSR) and Mindfulness-based Cognitive Therapy (MBCT) [16] and lack of condition-specific health self-management in manualised MBSR meant that participants felt that MBSR was not adequately adapted to MS or SCI [8, 14]. These barriers impede the uptake of mindfulness in populations in which mindfulness has been shown to have clear benefit for long-term self-management, by improving self-acceptance, goal attainment, social integration, and self-development [17]. Physically, therefore, adapting sensation-focused mindfulness-based practices to populations who have neurological injury is key to maximising engagement and health outcomes in such populations.

Psychologically, barriers encompass the significant challenges of approaching, during meditation and post-meditation enquiry, the reality of one’s health condition and its impact on identity [16]. Adoption of a fledgling mindfulness practice was also found to initially increase awareness of disability [18]. This may be as a result of introspection during meditation: renewing a focus on thoughts and emotions may increase the salience of the grief processes (such as denial, anger, bargaining, depression and acceptance) which are active after traumatic injury or chronic illness diagnosis [19–22]. Barriers may also be inherent in the willingness to engage with mindfulness at all as a practice due to insecurities surrounding perceived stigma associated with mindfulness and worries over efficacy [16]. These psychological barriers are held in tension with the clear research base suggesting that mindfulness may enhance variables associated with quality of life, from reduced stress [9], fatigue [3], depression and anxiety [8], to enhanced resilience [23] and self-compassion [24]. It is evident, therefore, that there is a need to consider the implications of engaging with mindfulness-based practices for populations with conditions associated with sensory loss. Developing clinical recommendations to adapt mindfulness for neurological injury should be a primary target to maintain parity in the availability and accessibility of mindfulness-based self-management techniques. The current study represents phase three of a programme of qualitative research which will support in the generation of clinical recommendations for teaching (adapted) mindfulness-based practices in populations with sensory loss. Phase 1 considered patient-perceived barriers to use of mindfulness after SCI [16] and Phase 2 addressed the impact of mindfulness on body awareness in expert meditators with neurological injury or impairment [25].

To fully explore the role of mindfulness in the management of symptoms associated with sensory loss, it is important to work with experienced mindfulness practitioners who have used mindfulness over a prolonged period of time whilst living with neurological disorders. As a result, this research aimed to explore how expert mindfulness meditators with neurological injury or impairment experienced using mindfulness for their own condition self-management, highlighting their rationale for doing so and the barriers that they perceived in their personal practice.

## Method

### Design

An inductive qualitative research design was used, with semi-structured interviews analysed via Interpretative Phenomenological Analysis [26].

### Participants and recruitment

The Breathworks National Registry of Mindfulness Teachers was used to recruit participants specialising in the delivery of Mindfulness for Health courses. Participants were purposively recruited [27] if they: had completed a formal mindfulness teacher training programme, taught three or more mindfulness courses per year, maintained an active personal mindfulness practice and had a formal diagnosis of a chronic health condition associated with neurological impairment causing sensory loss. Exclusion criteria were: historical mindfulness teacher training without ongoing teaching practice and a health status which did not include loss of sensation. Snowball sampling was employed to contact other eligible participants outside of this database [28].

Eight mindfulness teachers were recruited (five females, three males; Mean Age 52 ± 9.5 years). Participant demographic characteristics are contained in Table 1. All participants were wheelchair users, resident in the UK, and reported high levels of mindfulness above that of non-expert clinical populations on the Five Facet Mindfulness Questionnaire (see Hearn & Finlay [4] for comparison).

**Table 1:** Participant characteristics

Pseudonym	Age	Sex	Diagnosis	Date of diagnosis	Employment status	Teaching experience (years)	Daily practice (hours)	FFMQ					
								Observation	Description	Aware actions	Non-judgement	Non-reactivity	FFMQ (total)
Jade	35	F	FND	2012	Part-Time	3	1	38	40	34	36	32	180
Sarah	58	F	SCI (I)	1976	Full-Time	20 +	11	35	37	35	38	32	177
Pete	42	M	MS	2010	Part-Time	4	7	39	33	32	32	28	164
Keith	59	M	SCI (C)	1983	Unemployed	20 +	3	32	28	37	39	29	165
Robert	55	M	SCI (C)	1984	Part-Time	8	1	33	34	24	32	28	151
Tricia	46	F	SCI (I)	1995	Full-Time	10	3	32	20	30	38	25	145
Elise	59	F	SCI (I)	2009	Retired	20 +	10	38	35	32	35	32	172
Jenny	60	F	SCI (C)	1979	Volunteer	5	1	34	33	34	37	31	170
Mean	51.8					11.3	4.6	35.1	32.5	32.3	35.9	29.6	165.5
SD	9.5					7.7	4.1	2.9	6.1	4.0	2.7	2.6	12.2

FND = Functional neurological disorder; MS = multiple sclerosis; SCI (C) = complete spinal cord injury; SCI (I) =  incomplete SCI

## Materials

### Demographic questionnaire

Current health status, approximate date of diagnosis of SCI, MS or Functional Neurological Disorder (FND), formal mindfulness training history, teaching commitments and personal mindfulness practice were self-reported.

### Five facet mindfulness questionnaire (FFMQ; 29).

A 39-item measure of mindful non-reactivity, observing, acting with awareness, non-judgement and describing was used as a confirmatory indicator of expert levels of mindfulness. The FFMQ has demonstrated strong clinical applicability, consistency and reliability [30]. Items are scored on a scale from 1 = never to 5 = always true, with a maximum score of 195.

### Interview schedule

The authors developed a 9-item semi-structured interview schedule (see Table 2) in consultation with recommendations from an accredited mindfulness teacher and patient representative with SCI. The interview schedule was informed by preliminary qualitative research into the use of mindfulness in people with SCI [16, 25]. Interviews ranged from 50–93 min (mean duration 63.5 ± 16.87 min) and were conducted between May 2018 and September 2019.

**Table 2:** Interview Schedule

1. Can you tell me a little about how you first came across mindfulness?
a. How has your ‘journey’ developed since then?
2. Why have you continued to engage in mindfulness?
3. In your experience of SCI/MS/FND what role does mindfulness-based practice play in managing your condition?
4. Can you tell me about how you practice mindfulness?
a. How often?
b. Where?
c. When?
d. Which techniques?
5. How does your SCI/MS/FND impact upon your personal mindfulness practice?
6. What challenges to mindfulness have you encountered personally?
a. Physical
b. Emotional
c. Prejudice/stigma
d. Adapting techniques
7. How did you overcome these challenges?
8. Why do you think mindfulness is an effective approach for people with SCI/MS/FND?
9. This research has focused on the barriers and facilitators to mindfulness experienced by people with SCI/MS/FND. Is there anything more that you would like to tell me about?

### Procedure

Ethical approval was granted by the School of Psychology and Wellbeing Ethics Committee at the University of Buckingham and information about the research disseminated via the Breathworks Teacher Registry, with permissions. Interested participants were provided with a full copy of the information sheet and scheduled for an interview with the first author (KAF; n = 3) or a Trainee Health Psychologist (n = 5). Participants provided written, informed consent and completed the demographics questionnaire and FFMQ via email. All interviewers had completed advanced qualitative research methods and interview training as part of a BPS accredited postgraduate qualification and additional research project training including peer-reviewed role plays based on the interview schedule, discussions of interview delivery and fidelity (duration = 2 hours). Interviews were audio-recorded and transcribed verbatim, with identifying information anonymised. Transcription quality was audited by the first author. The semi-structured interview schedule was used to guide the interview, with participants encouraged to lead the interview through exploration of the issues most salient to their experiences [26], where appropriate. Interview schedule probes were used to further consider topics which were participant-generated but within the aims of the research [31]. Participants were debriefed, thanked for participation and given the opportunity to review original transcripts for validation and clarification if desired.

### Data analysis, quality and rigour

Idiographic analyses were undertaken to maintain a ‘bottom-up’ perspective, prioritising the experience of the participant. A reflexive log was maintained, enabling the ‘bracketing-off’ of assumptions and preconceptions, to minimise bias in interpretation [26]. After readings for familiarity, transcripts were analysed three-fold, for descriptive, linguistic and conceptual themes, which were clustered to develop emergent themes for each case, in association with the reflexive log. Cross-case analyses were recursively undertaken with the first author consistently moving between transcript and themes to ensure representativeness and integrity in the derived superordinate and subordinate themes. The themes presented are one possible interpretation of the data, respecting the interpretative nature of the analysis and the double-hermeneutic [32]. Full triangulation of the themes was completed by an auditor [JHH] with significant experience of IPA in clinical research. Disagreements were resolved through discussion with the third author [AC] and a process of constant comparison between themes and transcripts.

## Results

Analysis of transcripts resulted in two superordinate themes: (1) Overcoming a disrupted biography; and (2) Proactive self-management. Superordinate and subordinate themes and prevalence of themes within transcripts are shown in Table 3.

**Table 3:**
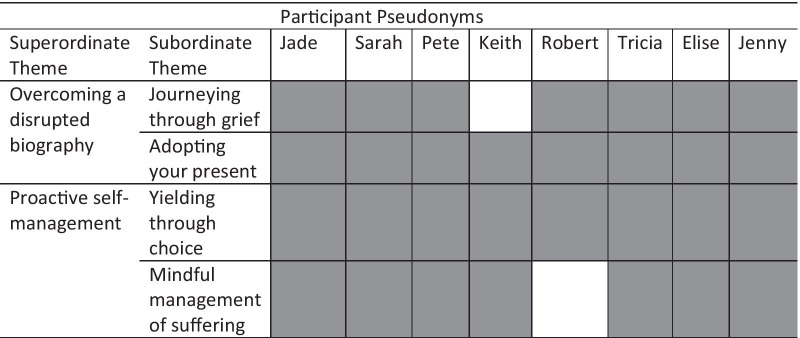
Superordinate and Subordinate themes and prevalence within transcripts

Key: Grey indicates theme was present in transcript

### Overcoming a disrupted biography

The first superordinate theme considered the weight of the challenge of adjusting to a ‘new’ identity after a significant change in health status due to sensory loss. Grieving the past self was normalised, allowing reconceptualization of one’s biography in the present moment, something which refocused perspective towards life beyond health restrictions.

### Journeying through grief

Adapting to life following chronic illness diagnosis or traumatic injury, was thought to initiate internal conflict between a desire for a return to the past and the struggle to adapt to a new health status:

I think it’d probably be a fairly generalizable truth for people who are living with anything that has given them a life changing impact on their functionality. Where that’s pain or whether that’s loss of mobility or, you know, whatever combination of those factors it is. I think it’s probably fairly generalizable to say that initially, you’re battling against kind of ‘I want to be what I was before’. The emotional difficulty with the mindfulness is that’s not going to give me what I had before. (Jenny)

Jenny normalises the challenge presented by this struggle as something widely experienced, irrespective of its physical cause. Its impact is directly on a fixed concept of a past identity, the desire to be a previous version of the self. Jenny is blunt in her discussion of the role mindfulness has in this battle: it is not a way of returning to the past, and therefore the emotions inherent in that struggle may require processing during mindfulness practice. Robert characterises this challenge further:

Obviously, there are going to be aspects of someone’s situation that they don’t want to be that way. They wish that it was different. When the mind gets hooked on wanting something to be different from how it is, it’s just a destructive loop of rumination. (Robert)

By the use of the term ‘hooked’, Robert is expressing the strength of the temptation to return to the past. He identifies this desire as something that occurs cognitively, in the mind, and that it is not benign, but potentially powerful and destructive. Sarah suggests that such a process may be related to grief processes active post-injury or diagnosis:

I think I’ve gone through all the stages of grief: denial, anger, bargaining, depression, acceptance. I think they’ve all been part of my journey and they’re all unavoidable. So, by turning towards what’s happening; what’s happening is I’ve got a serious injury and I’ve got a painful body then there’s bound to be difficult emotions tied up with that. And I’ve had to go through many phases of grieving and loss and anguish around all that. (Sarah)

For Sarah, accepting her SCI has been part of a journey, with the stages of grief as obstacles en route. Such a journey elicits emotional fallout and she has reached a stage in her grief process where she can approach her SCI, doing so through factual observation of her health status and realism about the inevitability of an emotional response. The intensity of this process is evident when she discusses the grief and ‘anguish’ she has been through. Yet by ‘turning towards what’s happening’, Sarah is suggesting that there is a need to responsively approach the body/emotions and associated grief. For both Sarah and Elise, it is clear that grief is to be worked with, rather than repressed:

I like to think of ways to pay tribute to the sorrow and to accompany it, but to move people through the doorway of that sorrow and myself too, so that I expand my world and my life in a different way. So, I think it’s important to recognize that grief can be a big barrier to a person reclaiming their life. (Elise)

Working with grief is therefore a necessity, as Elise sees it as a potential limiter of the ability to re-engage with living. Collectively, this theme demonstrates the significant challenge inherent in overcoming the desire to look backwards, and instead work with grief, accepting its emotional impact as unavoidable.

### Adopting your present

Moving past grief was considered to be specifically facilitated in mindfulness by acceptance and present moment awareness, core mindfulness techniques. Elise reflected upon how this occurred:

It [mindfulness] has helped me cope with many, many surgeries and months and months and months of recovery, where I felt trapped and to find the liberation away from that fear of being trapped. It has helped me live a fuller life where I focus more on this opportunity. I have this moment and it helps me keep some of those things more peripheral to my experience. You know, it’s like it is part [of] me and I manage it, but it’s not all of who I am. And so, what I am, who I am is where I’m at, at this present moment. And I don’t think I’d be doing as well as I am without mindfulness. (Elise)

Elise is able to use mindfulness to separate her health status from her self-concept. She has adopted her health restrictions as part of her, but not to the extent where she has become negatively enmeshed with this. She recognises that she lives in the present and that freedom from past and future fears is possible when she focuses on the now. Pete conceptualises this as part of accepting the fluctuations normal to the experience of MS:

For what I’ve got there’s no cure, you know, so there was lots of accepting; that’s a massive thing in mindfulness – accepting of the experience. I thought ‘this is it’, you know. It doesn’t make things any easier sometimes because I have days where it’s horrible, you know, but, yeah, I can just be with it. As you say in mindfulness, whatever it is, is already here. So that’s it. (Pete)

Pete recognises that acceptance is not just a single event, but is repeated, facilitated by mindfulness. Though he identifies some days with his MS as ‘horrible’, the tone of his final statement appears quite different to such emotional terminology: the level of his acceptance is shown by the very factual, almost unemotional recognition of the present as his reality. Pete’s depth of acceptance is also modelled in in similar comments by Keith, who describes his changed state of mind:

I guess the thing is that just by turning your mind to what is present, what’s going on, it starts to [pause]. Well, I suppose you start to resolve thoughts and feelings that you’re having. I think if you go about meditation or mindfulness in a useful, skilful way, then it just changes your state of mind and it changes it usually, in the long run anyway, for the better. It just seems to be inherent in doing that kind of practice that your state of mind becomes more open, more flexible, more sort of brighter. (Keith)

In this quote Keith expresses the power of staying present. For him, staying present has beneficial cognitive and emotional outcomes. That he mentions the need for skilful mindfulness practice indicates that these outcomes are not necessarily immediately evident, but instead may be the result of persistence. Jade terms such focused meditation as  ‘leaning towards your difficulties’:

The focus is really leaning towards your difficulties from kind of a self-compassionate place. When often you know you push them away, then opening up again and not just being so focused on your difficulties but allowing the rest of life experiences and then those little pleasant moments that we start to miss [to] come in and then opening up even further and then connecting to the world at large. (Jade)

Beginning with self-compassion, through meditation Jade is able to approach her health, which subsequently shifts her focus from introspection to observing elements of life which bring pleasure and foster social connectedness. This subordinate theme therefore reflects the power of present moment awareness within skilled mindfulness practice to bring acceptance of health and support in cognitive and emotional wellbeing.

### Proactive self-management

The second superordinate theme encompassed the decision to move towards accepting responsibility for living, surpassing the natural blame and anger which may occur post-injury/diagnosis. Mindfulness was felt to interrupt the relationship between illness and suffering, acting as a tool to prevent rumination and critical self-judgement and regain autonomy in health.

### Yielding through choice

Evident within all accounts was the importance of making an active choice during mindfulness practice in order to restore a sense of control in self-management of SCI/MS/FND. Diffusion of responsibility for health to others or healthcare providers was acknowledged to be a barrier to mindfulness:

There’s a lot of blame and then y’know, ‘if they hadn’t done this and they hadn’t done that’, ‘when are they gonna fix this’. So that was quite a barrier. Because in some ways I think certainly with people that have had an injury or are medically unwell, they can, you know, you can get very stuck in blame. And whilst you’re stuck in blame, you’re not being mindful. And it’s not taking responsibility for your own wellbeing. (Tricia)

Tricia is exemplifying some of the statements she has heard in the clients attending her mindfulness courses. She recognises that such statements risk the person remaining in a static position without the ability to move forwards towards regaining control of their health. The use of the word blame is potentially challenging here, bringing powerful suggestions of avoidant coping behaviours. Yet Tricia softens this by suggesting that blame instead represents being ‘stuck’, or stagnant in adjusting to injury/diagnosis. Elise suggests that this may be at times of heightened emotional load:

Regularly, I’ll see someone having a bad day where they’re caught up in their pain and they’re caught up in their panic and they’re caught up in their anger at maybe the surgeon who caused it or the doctor who injected their spine and caused it. (Elise)

It is in such a context of cognitive and emotional crisis that the role of choice was consistently presented by participants:

I always say to people you’ve got two choices. You can be in chronic pain and be miserable and have an awful life or you can be in chronic pain and have a good life. That’s your two choices. Going back to how you were before your injury, that’s not an option. That’s not on the table. (Sarah)

Here Sarah is laying out choice as having two outcomes: positive or negative quality of life. Inherent in her statement is recognition of the desire to return to the past, but the reality that such an option is not possible. It is in facing this choice that she feels mindfulness supports people in taking a step towards better quality of life:

We often say the behavioural outcome of mindfulness is choice. Through being aware, through knowing what you’re thinking, through knowing what you’re feeling in your body, through knowing what emotions are going on rather than just sort of having knee-jerk reactions to that, you insert a little bit of a gap and then you can make a choice. What’s going to be the most helpful response here? (Sarah)

Sarah finds that the heightened sensory, emotional and cognitive awareness that is promoted through mindfulness minimises reactivity and grows reflective space. In this space, autonomous choice can be reasserted, which is directly linked to implications for health and wellbeing. This can be facilitated in mindfulness practice by improving body awareness and non-judgemental observation of thoughts:

What we’re trying to do is to help them practice how they come into their bodies and step away from their minds that are spinning with thoughts so that they can be in that moment. So that they can take that pause, so that they can choose what they’re gonna do in the next moment. (Elise)

Through her experience in teaching mindfulness, Elise is promoting the reassertion of control over body and mind, enabling her students to claim the space which allows them to make a choice to manage their health differently. It is in this subordinate theme, therefore, that the impact of mindfulness in facilitating choice and self-management is clearly evident.

### Mindful management of suffering

Repeatedly discussed by participants was the concept of primary and secondary suffering. This was explained by Elise:

Like you have your primary suffering of the pain or the condition, but then you have that secondary suffering of all the thoughts and perceptions that you attached to that and it can grow, you know, like in a different process. And so, mindfulness has helped me to release attachment to that to where I guess I don’t worry as much about that potential future. (Elise)

Elise is separating suffering into two components: (1) biological/physical suffering experienced as a direct result of SCI/MS/FND; and (2) psychological aspects of suffering. Conceptualising suffering in this way forms part of teacher training advocated as part of Mindfulness for Health programmes. The implication for Elise is that she perceives that psychological suffering can be subjectively altered. Through mindfulness, she highlights her ability to reduce catastrophizing and minimise worry about her future self and health. Tricia also uses this framework to discuss how she can limit her responsiveness to pain using mindfulness:

It’s the secondary suffering. It stops the secondary suffering. So, it doesn’t necessarily stop the pain, but it stops the cycle of ‘oh God, I’m in pain and it’s a terrible day’. It’s keeping yourself grounded and just keeping a check on pain. Because widespread pain is more of a psychological issue than a physical issue so it’s kind of keeping a check on that. (Tricia)

That pain can be exacerbated due to psychological factors is something Tricia identifies as a cyclical risk. She feels it needs careful management, twice mentioning ‘checking’ this process, suggesting that it is the regularity of her daily mindfulness practice which facilitates her perceived capacity to control her subjective responses to her health. Sarah directly attributes self-management to mindfulness:

You’re going around with this primary plus resistance plus secondary which is a whole lot of suffering. And with mindfulness you can massively reduce the secondary suffering. ‘Coz in a way you’re doing that to yourself through your avoidant reactive responses. (Sarah)

Sarah is adding to the concept of primary and secondary suffering by interspersing resistance, in which you actively fight to resist your body and manage your health status (adaptively or maladaptively). She is direct in her assertion that you have the capacity to limit subjective reactivity and, therefore, suffering. Such direct language reflects the significance of the mindfulness practice for Sarah: it has facilitated self-management of her health, enabling her to reach a point where she is choosing to approach and take responsibility for, rather than avoid her condition. Keith considers this to be a form of ethics:

The whole things of ethics. Just realising that how I act and the states of mind that I allow myself to be a part of, have an impact upon myself and others. Sometimes more helpful, sometimes less. Sometimes, some states, they cause more suffering, some cause less. (Keith)

Keith’s use of the phrase ‘allow myself’, parallels the subjective assertion of control over one’s own health and suffering, presented by the other participants. In essence, all participants have demonstrated that through their mindfulness practice, they are proactively recognising their own role in their acceptance of their health condition and their self-management of potentially negative psychological outcomes.

## Discussion

Findings from the current study generated two superordinate themes; *Overcoming a Disrupted Biography* and *Proactive Self-management*, which together reflect the challenge of using mindfulness to facilitate self-management of health in conditions associated with sensory loss. The first superordinate theme foregrounded the significance of adjustment to injury/illness; the initial strength of desire to return to a past self (without sensory loss) was difficult to reconcile and move beyond. In desiring to return to a past without injury, grief processes were identified, particularly in terms of denial and anger. Mindfulness was used as a tool to manage this by preserving the ability to stay present and avoid rumination. However, mindfulness meditation was also considered to be a forum in which difficult emotions associated with injury/illness and the present self would need to be processed rather than avoided. From grief, and through skilled mindfulness practice, it was possible to accept one’s health status in spite of sensory loss, which fostered quality of life and social connection.

Grief following Spinal Cord Injury or chronic illness diagnosis has been widely explored, with individuals experiencing elevated grief and loss, reporting lower satisfaction with life [33]. The significance of grief processes after SCI has led to the development of a population-specific grief assessment tool [34] and a recognition that feelings of grief or loss can reduce participation in rehabilitation interventions [22]. That such a measure is needed indicates the urgent need to rationalise specific self-management strategies to enable patients to work through their distress and grief following SCI. The results of the current study add to the evidence supporting that grief is active in living with SCI/MS/FND and implicate mindfulness as a viable candidate for the self-management of such grief. Mindfulness was used to minimise the emotional impact and cognitive rumination associated with grief, for example during Kübler-Ross and Kessler’s denial and anger stages [21]. The current results demonstrated *active* self-management of grief, with participants reporting ‘turning towards’ their injury or illness within meditation practice. This exemplifies adaptive approach-based coping which can aid in the self-regulation of emotions and psychological wellbeing [35]. The current study demonstrated that by prioritising acceptance of the present, inclusive of one’s health status, psychological flexibility was enhanced, as also exhibited in chronic pain [36]. It is notable that such acceptance did not necessitate avoidance of negative physical/emotional/cognitive states – these were acknowledged as expected. Instead, mindfulness practice facilitated adaptation to illness/injury long-term, by promoting self-compassion, minimising rumination and worry about one’s past or future, and increasing engagement with valued activity [24].

The second superordinate theme identified the role of proactive self-management used to support the reconciliation of an individual’s past and present health status and foster day-to-day self-care. Principally this theme reflected the challenges inherent in regaining autonomy and a sense of responsibility over one’s own health status – factors considered core to successful rehabilitation and self-management [37]. With tightening rehabilitation timelines [38], self-management is increasingly important to facilitate the ability to take responsibility over one’s own health [39]. Mindfulness has been associated with condition self-management, self-efficacy and personal autonomy, resulting in improved psychological wellbeing and quality of life in both SCI and MS [40, 41]. The current study therefore confirms that mindfulness offers the opportunity for participants to explore and reflect upon their sense of autonomy, encouraging a progressive move towards condition self-management, an internal locus of control and better quality of life. This confirms the findings of Hearn, Finlay & Sheffield [17] who noted that the primary reason for engagement with mindfulness after SCI was that people felt that mindfulness was proactive and protective for their health. A tailored mindfulness-based self-management intervention, appropriate for those with sensory loss due to SCI/MS/FND, may therefore have strong potential for supporting future community health outcomes in these populations.

In the current study, participants perceived the subjective experience of suffering to be malleable, separating this into primary and secondary suffering [42], whereby the physical suffering caused by symptoms was differentiated from psychological responses, respectively. As a tool to manage ‘secondary suffering’, mindfulness was deliberately employed to relieve psychological distress associated with sensory loss and health decline [43]. The efficacy of mindfulness in the management of psychological distress has been widely evidenced in Multiple Sclerosis [3] and SCI [23]. Such psychological distress is common after SCI and MS, even up to twenty years after diagnosis [5], therefore is a key target for improving long-term outcomes for people living with sensory loss in the community [44]. Within the current study, results consistently demonstrated that mindfulness practice was used to *approach* difficult emotions, managing catastrophizing and worry. Given that rumination has been found to hinder acceptance in people living with chronic illness [45], this is an important finding. The qualitative themes within the current study did not suggest that mindfulness was acting to *stop* negative thoughts and appraisals, an avoidant process that has been linked with worsening depressive symptoms [46]. Instead, mindfulness supported practitioners in observing such thoughts without judgement, allowing them to review their thoughts and behaviours with self-compassion, promoting *proactive* choice and psychological flexibility rather than *reactive* catastrophizing and rumination. In the current study, participants linked this to an increased ability to recognise factors bringing them perceived pleasure (quality of life) and to engage in social interaction, both markers of psychosocial life engagement in SCI in response to heightened motivation and self-determination [47].

The current findings therefore demonstrated three processes active in mindfulness practice undertaken by experienced mindfulness teachers with sensory loss: i) the opportunity to approach (psychologically and physically) their past and present health status, symptoms and condition without judgement; ii) meditational practices including present moment awareness which allow (with practice) for a move towards self-compassion and acceptance of health in the present moment. Finally, iii) increased internal locus of control, autonomy, agency and self-efficacy, which facilitate proactive condition self-management rather than reactive cognitive states associated with early stages of grief.

## Limitations and future research

The current research used purposive sampling to recruit highly experienced teachers of mindfulness who lived with sensory loss. Whilst this sample offers detailed insight into prospective long-term benefits of mindfulness in such populations, the processes identified during their mindfulness practice may be limited to skilled practitioners and may have taken time to establish. Further research could aim to identify whether acceptance and psychological flexibility are widely experienced, irrespective of skilled or non-skilled mindfulness practice. Future research could also work with people with neurological injury or impairment who have discontinued mindfulness, as this would provide further valuable insight into barriers to mindfulness practice in this population. The current study also employed snowball sampling, which may have led to selection bias. However, the unique nature of the population characteristics sought for this study (experienced mindfulness teachers with neurological impairment/injury) meant that snowball sampling was an appropriate approach: snowball sampling is recommended for recruitment when participants are not easily accessible or are a part of a vulnerable group [48]. Due to the use of Interpretative Phenomenological Analysis, the themes identified in the current research represent only one possible interpretation of the data [26], however considerable effort has been made through careful reflexivity and analytical practice, to ensure that the results are strongly representative of participant experience. IPA has been criticised for potential weaknesses in ‘trustworthiness’ when working with data [49]. In an effort to maintain analytic trustworthiness [49], the research team conducted in-depth discussions about impressions and thoughts about the interviews, explicitly addressing presuppositions and implicit biases. A stance of reflective curiosity [50] was maintained throughout meetings and analysis in order to respect the double hermeneutic and reach informed, contexualised consensus.

## Clinical implications

Working with people with sensory loss through mindfulness, presents a specific clinical challenge. Previous research [25] has recommended adaptations to physical mindfulness practices such as body scanning and mindful movement for wheelchair users, but research has not considered the psychological impact. The current study demonstrates that experienced mindfulness practitioners with SCI/MS/FND, who are wheelchair users, qualitatively identify clear psychological benefits of mindfulness, including psychological flexibility, heightened present moment awareness, reduced catastrophizing and rumination. It is important to recognise the challenge demonstrated in this study of assimilating to a new health status following traumatic injury or chronic illness diagnosis; engaging in mindfulness may increase the salience of this process due to the introspective nature of meditation practice. This study highlights that sensitivity should be maintained when working mindfully in the context of sensory loss. It was clear that there was a need to allow space for exploration (and potential resolution) of health-related grief.

## Conclusions

In this study, mindfulness was experienced as psychologically supportive, enhancing *self-management* of health and *self-care* for experienced meditators with sensory loss, both factors found improve biopsychosocial outcomes for neurological populations [6]. Initial exposure to mindfulness was found to (re)activate trauma and grief processes, but these were resolved with practice and mindfulness was subsequently found to be highly beneficial for self-managing health. There is an urgent need to increase the repertoire of self-management techniques that can be used by patients with mobility and sensory limitations: parity should be evident in availability of self-management across health conditions, and the restricted ability to use standard mindfulness approaches with SCI/MS/FND means that the significant health benefits which mindfulness has demonstrated for other chronic conditions are not yet fully accessible. The benefits of mindfulness for experienced meditators with neurological injury or impairment are clearly evident and suggest that mindfulness is a strong approach for further use after SCI or MS/FND diagnosis.

## Data Availability

The datasets generated during and/or analysed during the current study are not publicly available due to the potentially identifiable nature of the targeted participant population but are available from the corresponding author on reasonable request.

## Abbreviations

BPS: British Psychological Society
FFMQ: Five Facet Mindfulness Questionnaire
FND: Functional neurological disorder
IPA: Interpretative Phenomenological Analysis
MBCT: Mindfulness-based cognitive therapy
MBSR: Mindfulness-based stress reduction
MS: Multiple sclerosis
SCI: Spinal cord injury
